# Acute Focal Bacterial Nephritis Caused by Staphylococcus simulans

**DOI:** 10.7759/cureus.31241

**Published:** 2022-11-08

**Authors:** Masato Shishido, Hiroki Kitaoka, Keiko Watanabe, Mayuka Fujimoto, Tadayuki Kumagai

**Affiliations:** 1 Department of Pediatrics, Yaizu City Hospital, Yaizu-shi, JPN; 2 Department of Pediatrics, Graduate School of Medicine, The University of Tokyo, Tokyo, JPN

**Keywords:** methicillin resistant, acute focal bacterial nephritis, acute lobar nephronia, coagulase-negative staphylococci, vesico ureteral reflux, staphylococcus sp, community-acquired urinary tract infection

## Abstract

A toddler girl presented to our hospital with a fever that lasted for five days. She had no prior history of urinary tract infections or contact with farm animals. Investigations revealed a diagnosis of acute focal bacterial nephritis (AFBN), and we initiated antimicrobial therapy with ampicillin and cefmetazole. On day five, methicillin-resistant coagulase-negative *staphylococci* were detected in her urine culture, and we changed the antibiotics to vancomycin. Antibiotic therapy was continued for 21 days, with no recurrence of fever. Finally, the bacteria were identified as *Staphylococcus (S.) simulans*, which is a common farm animal pathogen. Clinicians should be aware of the possibility of AFBN caused by *S. simulans*, even if the patient has no prior history of close contact with farm animals. If a rare organism is detected in urine culture during AFBN treatment, the patient should be treated with appropriate antibiotics for the pathogen.

## Introduction

Acute focal bacterial nephritis (AFBN), also known as acute lobar nephronia, is an acute, nonsuppurative renal infection with characteristic focal lesions observed on radiographic imaging [1]. It is regarded as a midpoint in the spectrum of acute pyelonephritis and renal abscess [1]. The main pathogenesis of AFBN is an ascending infection from the lower urinary tract [2]. It has been reported that 48% of children with AFBN have congenital anomalies of the kidney and urinary tract (CAKUT), such as vesicoureteral reflux (VUR), megaureter, and unilateral renal hypoplasia [2]. Although the most frequent pathogen of AFBN is *Escherichia coli*, *Staphylococcus* or *Enterococcus* species may also cause AFBN [2]. Reports of AFBN caused by pathogens other than these species are limited.

Here, we report a case of AFBN caused by *Staphylococcus (S.) simulans*, which is a rare human pathogen and common in farm animals. This case demonstrates how treatment for AFBN should be selected when rare organisms are detected from urine culture and the importance of searching for underlying diseases in AFBN.

## Case presentation

A toddler girl was referred to our hospital with a fever that lasted for five days. She had no prior history of urinary tract infections or fevers of unknown origin. On examination, she only showed fever and appeared to be in good condition, with no signs of upper respiratory infections, gastrointestinal diseases, or Kawasaki disease. She had no close contact with farm animals and had no previous medical history.

**Figure 1:**
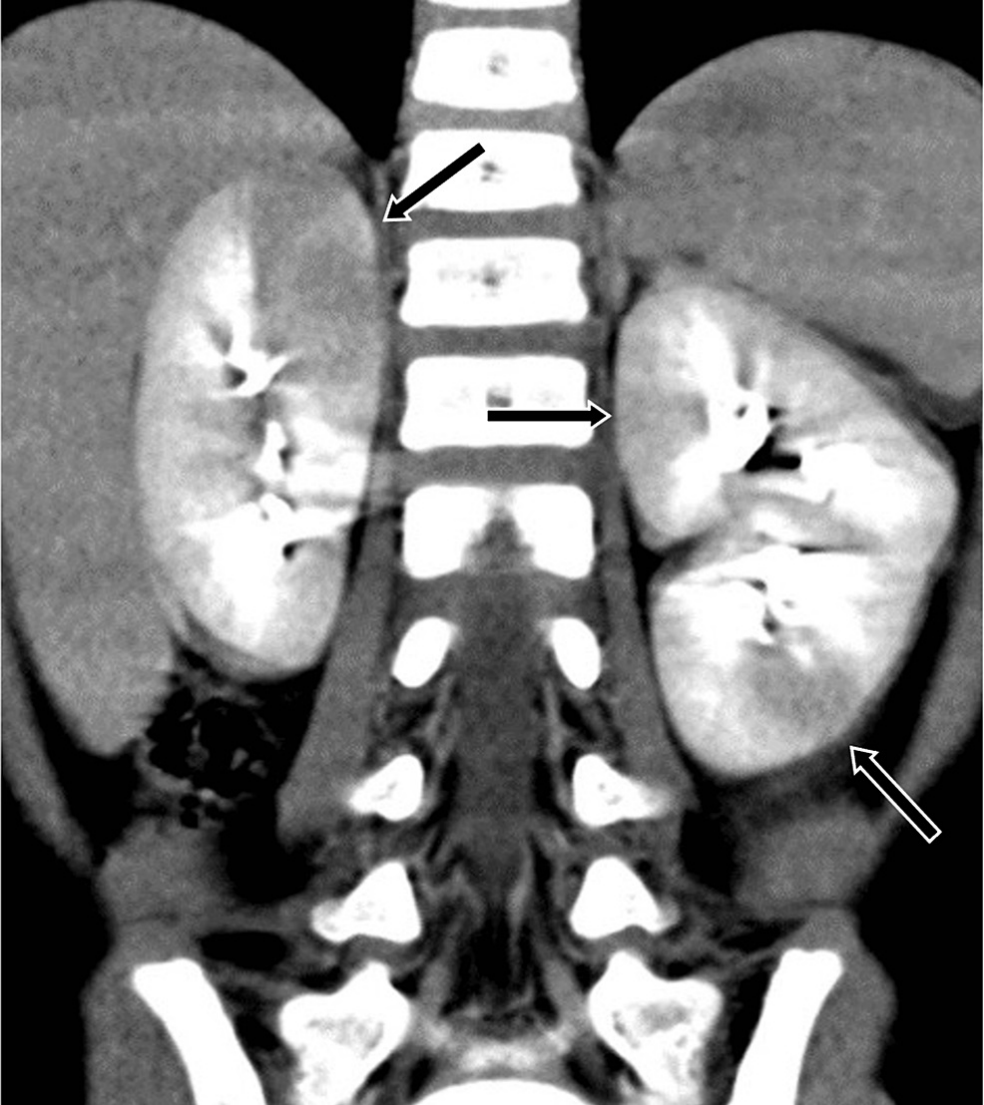
Abdominal contrast-enhanced computed tomography scan on admission Abdominal contrast-enhanced computed tomography (CT) scan on admission revealed multiple, wedge-shaped, low-density areas on both kidneys (arrows).

Laboratory tests revealed elevated inflammatory markers (white blood cell count 11,940 /μL and C-reactive protein 17.1 mg/dL). Although the catheterized urine test only showed a white blood cell count of 1-4 per high-power field, gram-positive cocci were identified using urine Gram staining. Since bacteriuria was detected, we suspected a urinary tract infection. An ultrasound of the kidneys revealed no abnormal findings. We suspected bacterial infections rather than viral infections because the inflammatory markers were considerably elevated. Although Kawasaki disease or bacterial infections, such as pneumonia or enterocolitis, were thought to be the differential diagnoses of fever, no signs of Kawasaki disease or gastrointestinal symptoms were observed, and chest radiographs showed no signs of pneumonia. To identify the source of the infection, a contrast-enhanced abdominal computed tomography (CT) scan was performed and revealed multifocal hypodense wedge-shaped space-occupying lesions in both kidneys, which are unique to AFBN (Figure 1).

A diagnosis of AFBN was made and the patient was treated with ampicillin (200 mg/kg/day) and cefmetazole (100 mg/kg/day) intravenously. Her fever subsided within 24 hours. On day five of admission, methicillin-resistant coagulase-negative s*taphylococci* were detected in her catheterized urine culture (1 × 10^5^ colony-forming units/mL). At this point, although the patient’s condition had improved and all physiological parameters had stabilized, the antibiotic was changed to vancomycin, to which the bacteria were susceptible. On day seven, the methicillin-resistant coagulase-negative *staphylococci* were identified as *Staphylococcus simulans*. The intravenous antibiotic was continued for 14 days, and then oral sulfamethoxazole/trimethoprim, to which the bacteria were also susceptible, was administered for seven days. There was no recurrence of fever, and the blood culture was negative throughout the course of treatment. Serum creatinine levels consistently remained within the normal range since admission.

**Figure 2:**
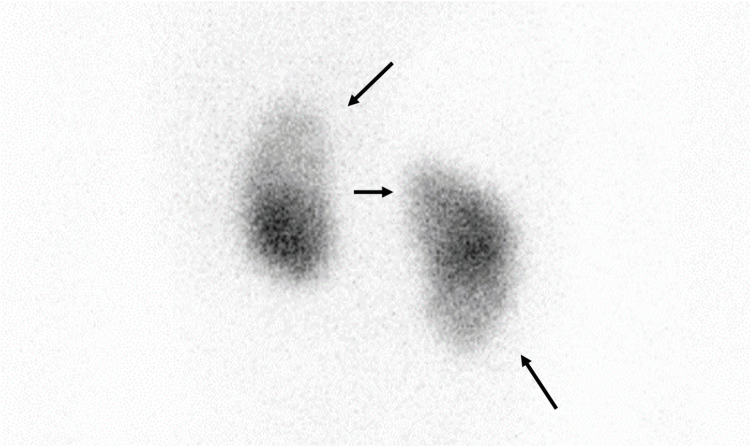
Technetium-99m dimercaptosuccinic acid renal scan after one month from onset Technetium-99m dimercaptosuccinic acid renal scan obtained after one month from the onset of the disease. There is a decrease in the renal uptake of radionuclides on both kidneys along the low-density areas in the initial CT scan (arrows).

**Figure 3:**
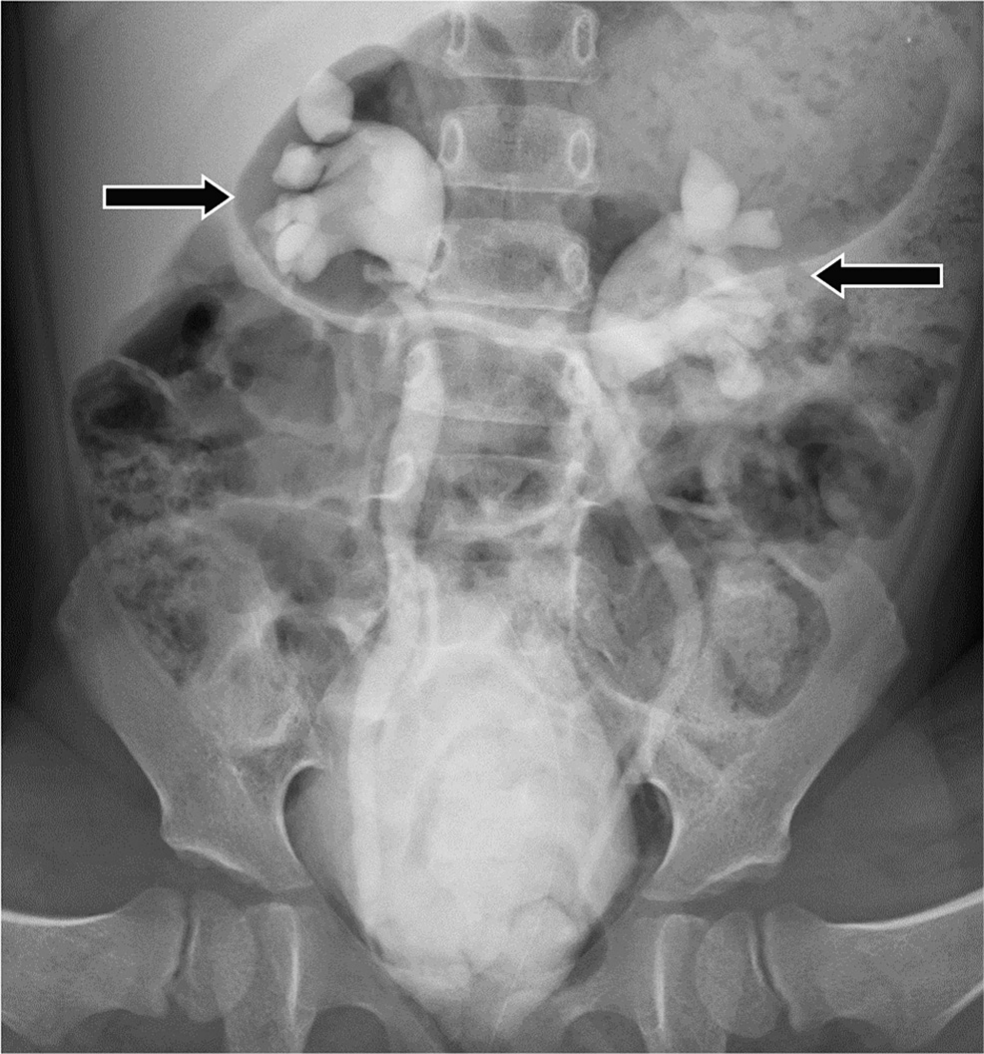
Voiding cystourethrography after one month from onset Voiding cystourethrography after one month from the onset revealed grade 3 bilateral vesicoureteral reflux (arrows).

One month after the onset of the fever, an ultrasound of the kidneys revealed no abnormal findings, and voiding cystourethrography (VCUG) and Technetium-99m dimercaptosuccinic acid renal scanning were performed. The results revealed renal scarring and bilateral grade 3 VUR, respectively (Figures 2, 3). Therefore, antibiotic prophylaxis with sulfamethoxazole/trimethoprim was initiated. No recurrence was reported in the eight-month follow-up.

## Discussion

In this case, the patient developed *S. simulans*-mediated AFBN and severe VUR was detected although she had no previous medical history. To the best of our knowledge, this is the first report of AFBN caused by *S. simulans* in children. *S. simulans*, coagulase-negative *staphylococci*, is a common farm animal pathogen that occasionally colonizes human skin [3]. *S. simulans* has been reported to cause soft tissue infections, endocarditis, bacteremia, osteomyelitis, prosthetic joint infections, and urinary tract infections [3]. Patients with *S. simulans* infections had often been in close contact with farm animals in previous reports [3,4]; however, several pediatric cases of *S. simulans* infection without exposure to farm animals have been reported [4,5]. Although our patient did not have a definite contact history with a domesticated animal, she sometimes did have contact with a neighbor’s dog. It has been reported that *S. simulans* can be present in healthy dogs [6], which may cause human infection through contact with pet animals. Additionally, in other studies, *S. simulans* was isolated in 5% of urethral urine samples from 100 healthy women [7], and *S. simulans* was isolated in 20% of urine cultures from 30 children with UTI caused by coagulase-negative *staphylococci *[5]. These studies support the hypothesis that *S. simulans* occasionally colonizes the human urethra not only through contact with farm animals but also through contact with common pets such as dogs. Although we could not identify the infection route in this case, contact with pet animals is potentially a risk factor for *S. simulans* infection.

We concluded that *S. simulans* was the main pathogen of the disease because the urine culture showed growth of more than 5 × 10^4 ^​​​​colony-forming units/mL without the presence of any other bacteria. Although the patient clinically improved after initial treatment, *S. simulans* in our patient was resistant to ampicillin and cefmetazole in the susceptibility test result. A multicenter retrospective study of 316 children with third-generation cephalosporin-resistant UTIs reported that most children experienced initial clinical improvement even if they initially received non-susceptible antibiotic therapy [8]. A possible explanation for this is that the antibiotics may reach higher concentrations in the urine than in the plasma and achieve a clinical response [9,10]. In addition, it was reasonable to start initial treatment with antimicrobial agents that did not target methicillin-resistant bacteria from the viewpoint of antimicrobial resistance. However, in terms of concerns regarding treatment failure and the perspective of antibiotic de-escalation, we adjusted the initially prescribed antibiotics to a set that targeted *S. simulans* infection for an additional 21 days. Switching to the appropriate antibiotics to which the etiological organisms are susceptible was recommended in a previous study [11]. Consequently, no bacterial recurrence was observed. Therefore, it is important to switch to appropriate antimicrobial agents when bacterial susceptibility is identified.

The recommended duration of AFBN treatment is a total of three weeks of intravenous and oral antibiotic therapy [12]. The optimal duration of intravenous antibiotic therapy is not established, but we selected two weeks of intravenous administration and one week of oral administration, expecting a robust treatment effect. Since it is suggested that intravenous antibiotic therapy can be shifted to oral form several days after the defervescence of fever [12], the hospital stay can be shortened depending on the patient’s clinical response.

Although the ultrasound of the kidneys one month later was normal, we performed VCUG to detect the presence of VUR, which revealed severe bilateral VUR. The gold standard investigation to diagnose and grade the severity of VUR is VCUG although it is an invasive procedure involving bladder catheterization via urethra and radiation burden [13]. Although renal ultrasound is not used for detecting VUR, abnormal ultrasound findings, such as dilatation of the renal pelvis or the ureter, increase in renal cortical echogenicity, and reduction in the thickness of renal parenchyma, are associated with VUR [14]. The other alternative procedure is voiding urosonography, but it needs intravesical administration of a contrast agent [13]. Although analysis of ureteric Doppler waveforms has been suggested as a noninvasive method to detect VUR, it cannot grade VUR [13].

We hypothesize that the patient had severe congenital VUR and developed AFBN caused by a rare pathogen. Coagulase-negative *staphylococci* have been reported as pathogens associated with UTIs, especially among patients with CAKUT, including VUR [5]. Since AFBN is also associated with a high rate of urinary tract malformations [2], VCUG should be performed in cases of AFBN caused by coagulase-negative *staphylococci*. When the presence of VUR is demonstrated, prophylactic antibiotic treatment or surgical correction should be considered for the prevention of another episode of UTI and the risk of further renal scarring [2]. VUR often resolves spontaneously [13]. However, high-grade VUR has a longer period to resolve and a lower resolution rate than low-grade VUR [13]. Long-term clinical consequences of post-infection renal scarring have been suggested to be associated with impaired kidney function, high blood pressure, and complications of pregnancy [13]. For appropriate treatment, clinicians should consider the possibility of CAKUT in cases of AFBN caused by rare pathogens.

## Conclusions

Clinicians should be aware of the possibility of AFBN caused by *S. simulans*, even if the patient has no prior history of close contact with farm animals. If a rare organism is detected in urine culture during AFBN treatment, the patient should be treated with appropriate antibiotics for the pathogen, and urinary abnormalities should be carefully investigated.
